# The decline of male sexual activity and function after surgical treatment for rectal cancer

**DOI:** 10.2340/1651-226X.2025.42015

**Published:** 2025-01-15

**Authors:** Anne Thyø, Peter Christensen, Ismail Gögenur, Marianne Krogsgaard, Michael B. Lauritzen, Birgitte S Laursen, Anette H. Mikkelsen, Asbjørn M. Drewes, Therese Juul

**Affiliations:** a The Danish Cancer Society Centre for Research on Survivorship and Late Adverse Effects, Aarhus and Aalborg University Hospitals, Denmark; b Department of Surgery, Randers Regional Hospital, Randers, Denmark; c Department of Clinical Medicine, Aarhus University, Aarhus, Denmark; d Department of Surgery, Aarhus University Hospital, Aarhus, Denmark; e Department of Clinical medicine, University of Copenhagen, Copenhagen, Denmark; f Department of Surgery, Centre for Surgical Science, Zealand University Hospital, Køge, Denmark; g Department of People and Technology, Roskilde University, Roskilde Denmark; h Department of Surgery, Aalborg University Hospital, Aalborg, Denmark; i Sexological Centre, Aalborg University Hospital, Aalborg, Denmark; j Department of Clinical Medicine, Aalborg University, Aalborg, Denmark; k Mech-Sense, Department of Gastroenterology and Hepatology, Aalborg University Hospital, Aalborg, Denmark

**Keywords:** Rectal cancer, male, sexual function, screening, late sequelae

## Abstract

**Background and purpose:**

The prevalence of sequelae following rectal cancer (RC) treatment is high. We investigate the prevalence and temporal change in sexual dysfunction among male RC patient, along with their counselling and treatment needs and associations between sexual dysfunction and clinical factors.

**Patient/materials and methods:**

Patient-reported outcome measures were completed 3 and 12 months after RC surgery. We used the five-item International Index of Erectile Function score to measure sexual function in sexually active patients and ad hoc items to explore their sexual activity level, causes of disrupted sexual life, and self-rated sexual function. Clinical data were obtained from the Danish Colorectal Cancer Group database

**Results:**

In total, 364 of 490 (74%) eligible male patients were included. Their mean age (standard deviation [SD]) at surgery was 68.3 (11) years. Forty-one percent reported being sexually inactive at the time of diagnosis. Among sexually active men, 44% had resigned from sexual activity at 12 months, mainly due to erectile dysfunction (ED), as reported by 55%. Only 16% experienced improvement; 19% experienced a worsening of their ED category in the 12-month observation time. Stoma was associated with both ED (odds ratio [OR] 5.6; 95% confidence interval [CI] [1.8, 17.4]) and low self-rated sexual function (OR 3.5 95% CI [1.8 , 6.7]). Phone contact to discuss sexual problems was requested by 29%; 19% were referred to professional treatment.

**Interpretation:**

Sexual dysfunction is common following RC, without improvement over time. Systematic screening enables identification of patients needing professional help.

## Introduction

Follow-up programs after treatment for rectal cancer (RC) vary worldwide. Their primary agenda is to detect cancer recurrence [1]. Recent years have seen a growing interest in late sequelae and their adverse impact on quality of life. Despite the high prevalence of organ-specific sequelae after RC [2–8], healthcare systems inadequately address these issues, leaving patients with unmet needs for help and treatment. In 2023, the Danish Cancer Institute conducted a cross-sectional study on over 3,000 cancer patients in Denmark, with 70% reporting sequelae impacting everyday life and 75% not receiving adequate help, especially concerning sexual and psychological sequelae [9]. Sexual dysfunction after RC treatment is well-described, especially in males. A systematic review and metanalysis including more than 9,000 male RC patients found that 1/3 of the patients had moderate to severe erectile dysfunction (ED), with no improvement within the first year after surgery [10]. Various treatments for ED are available [11, 12]; however, patients with problems must be identified. This study investigates a prospective cohort of Danish RC patients

We hypothesized that many patients experience persistent sexual dysfunction after RC treatment, with radiotherapy and stoma being key contributing factors. We aimed to investigate (a) changes in male sexual function between 3 months (3m) and 12 months (12m) after surgery for RC, (b) possible associations between sexual dysfunction and stoma/radiotherapy exposure, and (c) how many patients sought referral to professional sexological treatment.

## Patients/material and methods

Data for this study stem from an ongoing prospective cohort study systematically screening colorectal cancer patients for late sequelae and referring those needing specialized symptom management. Since 2018, patients have been invited to participate by completing patient-reported outcome measures (PROMs) at 3, 12, 24, and 36 months after surgery. Eligible patients received a link to the electronic PROMs via secure electronic mail (*eBoks*). Patients unwilling or unable to complete the PROMs online received a paper version. The REDCap (Research Electronic Data Capture) system was used for data collection and management [13]. RC patients were sent PROMs to check their bowel function or stoma function, urinary function, sexual function, pain, and quality of life, whereas colon cancer patients were sent PROMs to check for bowel- or stoma function and quality of life. Detailed study methods have previously been described [14]. This study focused merely on males treated for RC, with PROMs completed at 3m and 12m after surgery

Inclusion criteria were males undergoing surgical resection for RC with or without neoadjuvant/adjuvant treatment. Exclusion criteria were age under 18 years, linguistic limitations, cognitive impairments, local excision (i.e. trans anal and endoscopic mucosal resection [EMR]/polypectomy techniques), and advanced cancer requiring pelvic exenteration or multiorgan resection

The PROMs were revised in June 2020 to include items related to sexual activity and causes of disrupted sex life. Therefore, patients who completed the 12m PROMs before this time point were excluded. Patients treated in Aarhus and Aalborg were included from 20 June 2019 to 31 December 2022. As the surgical center in Koege started inclusion later, patients included from that specific center were treated between 1 April 2021 and 31 December 2022.

### PROMs

The validated five-item International Index of Erectile Function (IIEF) questionnaire [15, 16] was used for evaluating sexual function, with response options ranging from 1 to 5 points (very low, low, moderate, high, and very high). Patients were categorized into five groups of ED: severe ED (5–7), moderate ED 8–11, mild to moderate ED (12–16), mild ED (21–17), and no ED (22–25). A cut-off value of 21 points provides a sensitivity of 98 and a specificity of 88 for diagnosing ED patients [17]. Several IIEF items address intercourse making them irrelevant for sexually inactive patients. The IIEF total score was presented to and calculated only for sexually active patients. However, item 1 (‘*How do you rate your confidence that you could get and keep an erection?*’) and an *ad hoc* item ‘*How would you rate your sexual function during the past 4 weeks*’ were analyzed for both sexually active and sexually inactive men.

The question about sexual activity was obtained at 3 months follow-up, asking about activity level with response-options; (a) *‘I was not sexually active before diagnosis and have not commenced sexual activity since’*, (b) *‘Since my diagnosis, I have resigned from sexual life’,* or (c) *‘I am still sexually active’.* Patients responding to option (a) were not investigated further.

The PROMs also included one item: ‘*Do you wish to be contacted to discuss treatment options for any late sequelae’*? At patient request, a healthcare professional contacted him within 2 weeks with detailed information and referral options for professional counselling. Patients requesting further assistance were referred to the ‘*Clinic for Treatment of Sexual Dysfunction after Pelvic Organ Cancer*’ at Aalborg and Aarhus University Hospitals, which are independently operated by nurses trained in clinical sexology.

Demographic and clinical data were obtained from the Danish Colorectal Cancer Group’s (DCCG) database, while oncological treatment and stoma reversal timing were extracted from the medical records. The DCCG collects data prospectively with very high completeness. It comprises data about patients’ demographics, tumor characteristics, disease stage, and intra- and postoperative course.

### Statistics

Patients were excluded from the analyses if no sexual items were completed at either 3m or 12m follow-up. Furthermore, tardy responses were excluded, defined as 3m PROMs completed later than 6 months after surgery and 12m-PROMs completed later than 16 months after surgery.

Normally distributed, continuous data were presented as means and standard deviations (standard deviation [SD]); non-normally distributed data, as medians and interquartile ranges (IQR). Categorical data were presented as numbers and percentages. Paired Students *t*-test or Wilcoxon signed rank test was used to compare 3m and 12m PROMs, depending on the distribution. Change-over-time analyses were performed using percentage change analyses.

Logistic regression analyses investigated the association between ED and clinical variables. Likewise, the association with self-reported insufficient sexual function was investigated. The five ED categories were dichotomized into ‘no ED’ and ‘some degree of ED’, with a cut-off point of 21. Likewise, the response categories to ‘How do you rate your sexual function the past 4 weeks’ were dichotomized into ‘very good/good/acceptable’ and ‘bad/very bad’. Twelve-month responses were used for these analyses to ensure adequate distance from treatment, assuming a more stabilized sexual function. Potential confounding variables were adjusted for in the analyses, using a maximum of one variable for every tenth event. Data management and analyses were performed using Stata version 18.0. A *p* ≤ 0.05 was considered statistically significant

## Results

### Patient characteristics

**Figure 1:**
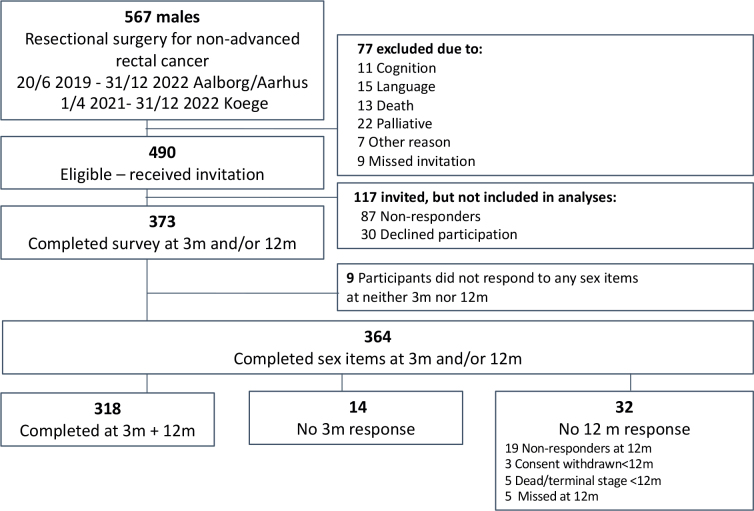
Flowchart for inclusion of patients.

A total of 490 patients were eligible for inclusion, and 364 responded to at least one item about sexual function at 3m and/or 12m (inclusion-rate = 74.3%) (Figure 1).

Baseline characteristics are shown in Table 1. Among patients who had a low anterior resection, 119/181 had a temporary diverting stoma during primary cancer surgery. It was not yet reversed in 103 patients at 3m and in 20 patients at 12m

**Table 1:** Baseline patient characteristics (*n* = 364).

Patient demographics, baseline	
**Age at surgery, years**	
**Mean (SD)**	68.3 (11.0)
**Range**	33–92
**ASA,** *n* **(%)**	
I–II	289 (79.4)
III–IV	75 (20.6)
**BMI,** *n* **(%)**	
< 25	136 (37.4)
>/= 25	228 (62.6)
**pT-stage, *n* (%)**	
pT0	5 (1.4)
pT1/pT1	35 (9.6)
pT2/pT2	103 (28.3)
pT3/pT3	205 (56.3)
pT4/pT4	11 (3.0)
missing	5 (1.4)
**pN-stage, *n* (%)**	
pN0/pN0	227 (62.4)
pN1/pN1	102 (28.0)
pN2/pN2	30 (8.2)
missing	5 (1.4)
**pM-stage, *n* (%)**	
pM0	342 (94.0)
pM1	22 (6.0)
**Procedure, *n* (%)**	
Low anterior resection (LAR)	181 (49.7)
TME	114
PME	67
Abdominoperineal resection (APR)	167 (45.9)
Hartmann’s procedure	16 (4.4)
**Surgical access, *n* (%)**	
Laparoscopy	113 (31.0)
Robot-assisted	228 (62.7)
Open	23 (6.3)
**Neoadjuvant short/long course radiotherapy, *n* (%)**	93 (25.5)
**Neoadjuvant chemotherapy, *n* (%)**	60 (16.5)
**Adjuvant chemotherapy, *n* (%)**	69 (19.0)

SD: standard deviation; ASA: ; BMI: body mass index; TME: ; PME: .

### Change in sexual activity between 3 and 12 months

**Figure 2:**
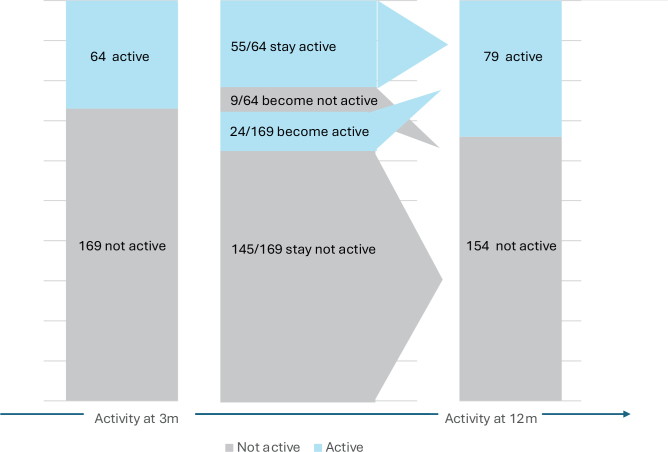
Change in sexual activity from 3 to 12 months (*n* = 233).

The sexual activity item was answered at both 3m and 12m by 233 patients, 169 of whom (73%) reported being sexually inactive at 3m (either inactive before diagnosis or having resigned after diagnosis), while 64 (27%) were active. Among the 169 inactive patients, 24 (14.2%) had resumed sexual activity by 12m. Conversely, of the 64 patients who were sexually active at 3m, 9 (14.1%) were no longer active at 12m. However, most patients *n* = 200 (85.8%) maintained their activity level over time (Figure 2)

### Sexual activity and causes of a disrupted sexual life

**Figure 3:**
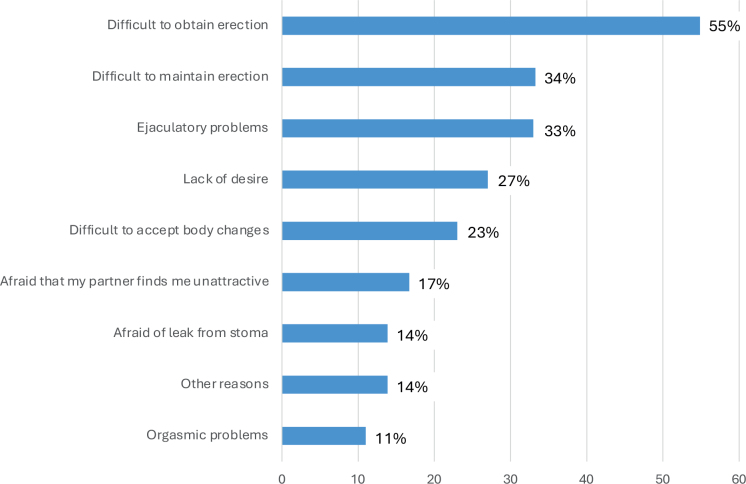
Causes of disrupted sexual life at 12 months, % (*n* = 83).

**Figure 4:**
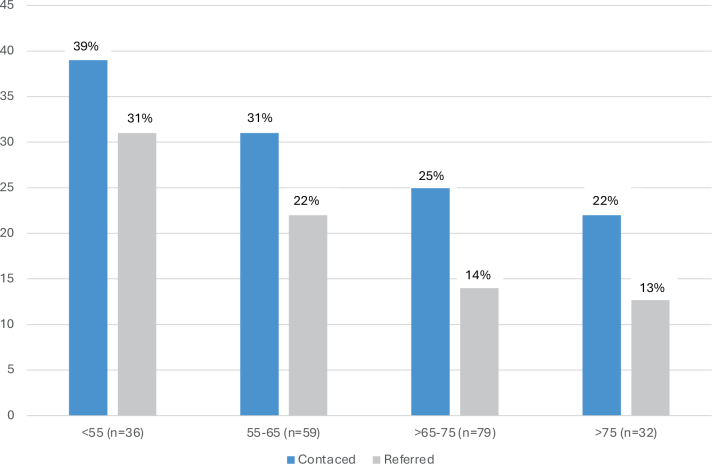
Telephone contact regarding sexual problems and referral to sexological treatment in different age groups (only patients sexually active at the time of diagnosis [*n* = 206]).

At 12 months, 319 patients responded regarding their sexual activity level. Of these, 131 (41%) were inactive before diagnosis and remained so. Among the 188 patients who were sexually active at diagnosis, 83 (44.1%) had resigned from sexual activity at 12 months (Figure 3). The reasons for disrupted sexual life among those who had resigned at 12m are presented in Figure 4, with patients being able to select multiple reasons.

The most common reasons for resigning from sexual life were problems with obtaining 46/83 (55.4%) and maintaining 28/83 (33.7%) an erection. Lack of desire (22/83, 26.5%) and body image issues (19/83, 22.9%) were also prevalent

### Change in ED scores from 3 to 12 months

A total score could be calculated for 89 patients at 3m; for 97, at 12m. However, only 69 patients responded to all five IIEF items at both time points, allowing for paired analyses. We found no difference in IIEF score from 3m to 12m, (19.9 95% CI [18.6, 21.2] vs. 19.7 95% CI [18.5, 21.0], *p* = 0.64)

From 3m to 12m, 45 (65.2%) patients remained in the same ED group, 13 (18.6%) worsened, and 11 (15.7%) improved. At 3m, 50/89 (56.2%) had an IIEF5 score < 21, indicating ED, compared with 53/97 (54.6%) at 12m

### Change in sexual confidence

The item ‘*How do you rate your confidence that you could get and keep an erection*?’ was answered by 250 patients at both time points. At 3m, 111/250 (44.4%) rated their confidence as low/very low; at 12m, 122/250 (48.8). Confidence remained unchanged in 140 (56%) patients, decreased in 64 (25.6%), and increased in 46 (18.4%).

### Change in self-rated sexual function

The item ‘*How would you rate your sexual function during the past 4 weeks?*’ was answered by 250 patients at both time points. At 3m, 53 patients responded, ‘*I don’t know*’ and were excluded from paired analyses; at 12m, 42 patients reported so. At 3m, 91/176 (51.7%) rated their sexual function as bad/very bad; at 12m, 85/176 (48.3%). Sexual function remained unchanged in 125 (71.0%) patients, worsened in 26 (14.8%), and improved in 25 (14.2%)

### Risk factors for ED and low self-rated sexual function

The ED categories and the self-rated sexual function responses were dichotomized to conduct logistic regression analyses. At 12m, *n* = 97 had completed the IIEF, where 53/97 (55%) had ED. The item ‘*How do you rate your sexual function the past 4 weeks*?’ was completed by *n* = 292. Patients responding, ‘*I don’t know*’ (*n* = 57) were excluded from analyses. A total of 131/235 (56%) reported bad/very bad self-rated sexual function. We hypothesized that radiotherapy and stoma are important factors associated with ED. Among the 97 patients, 22 (22.7%) had radiotherapy and 31 (31.9%) had a stoma. In crude analyses, the odds ratio (OR) for ED was 2.7 95% CI [1.0, 7.8] for radiotherapy exposure; 5.7 95% CI [2.0, 15.6], for stoma exposure. When applied to a logistic regression model with stoma/radiotherapy, age and American Society of Anesthesiologist (ASA) score added as adjusting variables, only stoma presence remained a statistically significant risk factor (OR = 5.6; 95% CI [1.8, 17.4]) (Table 2). Analysis of self-rated function encompassed 235 patients: 59 (25.1%) had radiotherapy and 116 (49.4%) had a stoma. In crude analyses, the OR for low self-rated sexual function when exposed to radiotherapy was 2.0 95% CI [1.1, 3.7]; for stoma, 4.4 95% CI [2.5, 7.6]. In the regression model with stoma/radiotherapy, age, ASA score, and Body Mass Index (BMI) added as adjusting variables, stoma presence remained a statistically significant risk factor, OR = 3.5 95% CI [1.8, 6.7] (Table 2). BMI > 25 was also nearly significant with OR = 2.0 95% CI [1.0, 3.7]

**Table 2:** Odds ratios for erectile dysfunction and low sexual function.

*n* = 97	No erectile dysfunction, *n* (%)	Erectile dysfunction, *n* (%)	Crude OR	Adj. OR*
Radiotherapy				
No	38 (39.2)	37 (38.1)	1 (ref)	1 (ref)
Yes	6 (6.2)	16 (16.5)	2.7 [1.0;7.8]	1.5 [0.5;5.1]
Stoma				
No	38 (39.2)	28 (28.9)	1 (ref)	1 (ref)
Yes	6 (6.2)	25 (25.7)	5.7 [2.0;15.6]	5.6 [1.8:17.4]

*n* = 235	Good self-rated sexual function, *n* (%)	Low self-rated sexual function, *n* (%)	Crude OR	Adj. OR**

Radiotherapy				
No	85 (36.2)	85 (36.2)	1 (ref)	1 (ref)
Yes	19 (8.1)	19 (8.1)	2.0 [1.1:3.7]	1.3 [0.6;2.8]
Stoma				
No	73 (31.0)	46 (19.6)	1 (ref)	1 (ref)
Yes	31 (13.2)	85 (36.2)	4.4 [2.5;7.6]	3.5 [1.8; 6.7]

OR: odds ratio

* Adjusted for radiotherapy/stoma, age, ASA score.

** Adjusted for radiotherapy/stoma, age, ASA score, and BMI.

### Phone contact and referral to treatment

Patients’ requests for phone contact and treatment referral were explored separately for patients who were sexually active at diagnosis and those who were not. We categorized 351 patients as +/- sexually active at the time of diagnosis based on their response at 12m (*n* = 319). In case of no response at 12m, the response at 3m was used (*n* = 32). Thirteen patients were not categorized as they did not respond to the item at any event. A total of 206 were sexually active and 145 were inactive at the time of diagnosis.

Among the sexually active men, 59/206 (28.6%) requested phone contact with a nurse to discuss their sexual problems and treatment options; 39/206 (18.9%) were referred to professional counselling and treatment. Younger patients (<55 years) had a high need for help, with 39% requesting phone contact and 31% being referred for treatment (Figure 5)

Patients requesting contact were generally younger, with a mean (SD) age of 62.6 (12.1) versus 65.9(10.5) (*p* = 0.05). Likewise, those who requested referral for sexual dysfunction treatment were younger (mean age 61.1, SD = 12.9) than those who did not (mean age 65.8, SD = 10.4) (*p* = 0.02). Still, even in the oldest age group (> 75 years), some patients requested help with sexual problems (Figure 5). Among the 145 sexually inactive patients at diagnosis, only 13 (9.0%) requested contact regarding sexual problems; 6 (4.1%) were referred to treatment.

## Discussion

These results demonstrate the need for attention to male sexual function after RC treatment. Nearly half of those sexually active before diagnosis had resigned from sexual life after one year, mainly as a result of erectile difficulties. Lack of sexual desire and body image issues were also important reasons. Among those still sexually active, more than half reported some level of ED. This study suggests limited improvement in sexual function within the first year after surgery.

### Prevalence and impact of ED

Among sexually active patients, 55% had ED. Previous studies have reported similarly high rates, with prevalences of 54–87% [10, 18–20]. A newly published systematic review investigated ED after RC treatment. In total, 9,006 patients were included. The study reported a prevalence of moderate–severe ED in 35% of the patients within the first year after surgery, using the IIEF-5/IIEF-15 score [10].

In unadjusted analyses, we found that radiotherapy and stoma were associated with ED, which is echoed in a previous retrospective cohort study of Danish male colorectal cancer patients [20]. However, only stoma was significantly associated with ED when adjusting for other variables. Since most stoma patients at 12m had an abdominoperineal resection (APR), this study cannot determine whether ED was caused by the procedural dissection or the stoma itself, given the stoma’s association with altered body image and sexual self-image [21, 22]. A study from 2020 on Danish and Swedish RC patients [23] found that a stoma was associated with sexual inactivity at 12m after treatment ended. Other studies have shown that patients undergoing APR have higher sexual dysfunction rates [24, 25] than those undergoing lower anterior resection (LAR). The psychological impact of a stoma may not directly affect erectile function. Still, both desire and erectile function may be affected by low self-esteem derived from altered body image and worries about not being attractive. Confirming this, regression analysis showed that a stoma was highly associated with low self-rated sexual function. In the adjusted analyses, we found no clear association between radiotherapy and ED or low self-rated sexual function. However, in previous studies, radiotherapy has been found to negatively affect erectile function [26, 27, 28]. As mentioned earlier in the text, ED was the main cause for disruption of sexual life among those who were active at diagnosis. With treatment for ED, it should be possible to restore sexual life

### Other sexual problems

Most studies of male sexual dysfunction after RC treatment focus on ED and ejaculatory dysfunction [20, 29, 30]. However, sexuality is multifaceted and individually perceived. Body image, relationship, and intimacy are also important factors [31] but have previously been studied only in female RC patients [32, 33]. Lack of desire, body-image problems, and concerns about not being attractive were reported by 17–27% as reasons for resigning from sexual life, highlighting their importance for males as well. Studies have shown that healthcare professionals are reluctant to initiate conversations on such issues. Hence, a study from 2014 described how patients found it embarrassing or inappropriate to discuss sexual matters. Clinicians face barriers such as inadequacy and inappropriateness, lack of professional training, and fear of raising irrelevant issues given the patient’s age and relationship status [34].

### Awareness and identification of sexual dysfunction

Clinicians are obliged to inform patients about potential adverse treatment side effects and to follow-up. However, Hendren et al. found that only 9% of RC patients recalled discussing sexuality before treatment [35]. Another study reported that most patients valued discussing sexual matters with their clinician and preferred the clinician to initiate the conversation [36]. As presented here, systematic screening using electronic PROMs may overcome these barriers.

We found that younger patients had a greater need for telephone contact and referral for professional treatment. Still, older patients were also referred, showing that the importance of sexuality should not be age discriminated against. Sexuality is an essential quality-of-life dimension, especially for young patients due to its long-term consequences.

First, patients must be identified. We believe systematic screening via electronic PROMs effectively identifies patients with sequelae needing treatment. Electronic PROMs allow patients to report issues easily and receive guidance or referral, breaking down barriers with healthcare professionals. This approach can reduce unmet needs and problems, and early intervention may prevent worsening over time. Previous strategies have been tested, including patient-led follow-up in the ‘Follow Up after Rectal Cancer Trial’, where patients were randomized to standard cancer follow-up or patient-led follow up. Patients in the intervention arm participated in an educational session regarding late sequalae and were encouraged to contact a specialist nurse in case of any symptoms or concerns. The study found no difference in prevalence of any late sequelae between the intervention- and the control arm and patients with sexual problems did not refer themselves or contacted the nurse with these problems [37, 38]. This underlines the need for out-reach to the patients; however, this must be balanced since not all patients have problems or the need to discuss them.

### Strengths and limitations

This is, to our knowledge, one of the largest prospective studies on sexual dysfunction in male RC patients, with 364 patients and a high inclusion rate of 74%, surpassing other studies of sexual dysfunction. The main strength of this study was its prospective design and the remarkably high response rate. Data were collected at different proximate time points after surgery, minimizing the risk of recall bias, featuring short, validated questionnaires and a few *ad hoc* items to minimize the item burden. All these factors contribute to a high level of external validity. This study also adds knowledge and quantification of male RC patient’s reasons for quitting sexual life and enumerate their need for help and counselling. Similar records have not previously been published. Approaching patients with systematic screening and enabling patients to request a phone call for information about any late sequelae, is unique. Screening with e-PROMs avoid face-to-face contact, facilitating more accessible and approachable contact and referrals, reducing potential embarrassment. Another strength is, that in our sexological clinics, treatment is typically based on a bio-psychosocial approach. It involves not merely medical treatment, but also guidance in the use of sexual aids, couples therapy, and individual talk therapy, covering all aspects of sexual dysfunction.

No baseline data on patients’ declining participation was available, therefore no analyses comparing responders to non-responders was conducted. Selection bias might have affected our results’ generalizability if the included patients differed markedly. However, their characteristics matched the average Danish RC patient per the 2018 DCCG clinical report [39], limiting the risk of selection bias.

We lacked pre-treatment sexual function data, only having activity information as reported at 3m follow-up. This could introduce recall bias, however unlikely, since patient ought to remember only 3m back in time. Pre-treatment evaluation is relevant, especially among older patients as sexual dysfunction may have existed before treatment. A large population-based Danish study from 2018 (the SEXUS project) investigated sexuality in a random sample of 62,000 Danes aged 15–89 with a response-rate of 34.6%. Overall, 27% of males reported ED. However, ED prevalence increased markedly in patients over 70 [40]. Certainly, we cannot determine if ED in older patients existed before diagnosis or was induced by treatment. However, most IIEF respondents were sexually active before diagnosis, assuming some erectile capacity. Generally, background information on sexual function in the general Danish population is lacking. The SEXUS project is the only existing data, however a response-rate of only 34.6% jeopardizes the generalizability. However, obtaining pre-diagnostic data about sexual functioning can be unreliable as patients are likely affected both physically and mentally by their diagnosis.

Finally, isolating and adjusting for all possible confounders in a logistic regression analysis was challenging. Firstly, the analyses included a relatively small number of patients. Secondly, multiple possible confounders exist, including body image issues, depression, fatigue, and the psychological impact of cancer treatment. Those variables are complicated to include in analyses and may profoundly affect ED and self-rated sexual function. In the analyses, we combined neoadjuvant and adjuvant chemotherapy into one variable. Neoadjuvant treatment is linked to higher cancer stages and may be associated with having a stoma. This may partly explain why radiotherapy was not statistically associated with ED but with stoma.

### Perspectives

The systematic screening for late sequalae was recently implemented as part of the standard clinical follow-up program at one of the including centers (Aarhus). After the implementation, an increase in the response rate has been observed, meaning that an even higher proportion of the future patients will be screened for late sequelae. Also, we are currently working on implementing a mobile application (an app) containing a digital care guide which informs patients about their treatment and follow-up pathway, common late sequalae, and how to manage them. One of the aims is to ensure that all patients are sufficiently informed about common late sequelae, here among sexual dysfunction, and that those with a need are identified and offered treatment throughout their entire follow-up pathway.

## Conclusion

Despite optimized minimally invasive, nerve-sparing surgery generally practiced in Denmark today, sexual dysfunction in male RC patients is still a common occurrence. Also, this study found it to be persistent within the first year. A stoma is associated with both ED and low self-rated sexual function. The need for help was highest among the younger patients who were sexually active before diagnosis.

Sexual dysfunction can be challenging to detect as many patients and clinicians are uncomfortable discussing it. To address this, we used PROMs to systematically identify patients with sexual dysfunction and refer those in need to professional treatment. This systematic approach is superior and more effective than previous tested strategies identifying a high number of patients with late sequelae, securing that those in need are managed professionally. Systematic screening should be implemented in future RC follow-up programs to improve sexual function over time.
